# Severe mitral stenosis masquerading as cardiogenic shock successfully managed with extracorporeal membrane oxygenation and percutaneous mitral commissurotomy: a case report

**DOI:** 10.1093/ehjcr/ytad553

**Published:** 2023-11-07

**Authors:** Syed Jalil, Ashraf Ahmed, Mahmoud Abdalla, Mohammed Al-Hijji

**Affiliations:** Weill Cornell Medicine, College of Medicine, Qatar Foundation, Doha, Qatar; Internal Medicine Department, Hamad Medical Corporation, Doha, Qatar; Department of Cardiac Anesthesia, Hamad Medical Corporation, Heart Hospital, Doha, Qatar; Weill Cornell Medicine, College of Medicine, Qatar Foundation, Doha, Qatar; Department of Structural Cardiology, Hamad Medical Corporation, Heart Hospital, Doha, Qatar

**Keywords:** Cardiogenic shock, Rheumatic valvular disease, Mitral stenosis, Percutaneous mitral commissurotomy, Mechanical support, Case report

## Abstract

**Background:**

Rheumatic fever is still a major cause of mitral valve (MV) stenosis in the developing world. Few patients with critical rheumatic MV stenosis can present with acute cardiogenic shock (CS) that requires urgent treatment with circulatory support and definitive valvular repair or replacement.

**Case summary:**

A 37-year-old gentleman was admitted with heart failure, CS Society for Cardiovascular Angiography and Interventions D, and atrial fibrillation with a rapid ventricular response. He had no prior medical history. He had multiple organ failures and required intubation, two DC shocks of 200 joules without haemodynamic improvement, continuous renal replacement therapy, and medical and mechanical circulatory support using extracorporeal membrane oxygenation (ECMO). His echocardiography showed severe rheumatic mitral stenosis (mitral valve area 2D of 0.7 cm^2^, mean diastolic gradient of 17 mmHg, Wilkins score 7). His Society of Thoracic Surgery score and EuroScore were 50.1% and 12.1%, respectively. Thus, a percutaneous transcatheter mitral commissurotomy (PTMC) was decided as the definitive treatment in a multidisciplinary team meeting. Following the procedure, the patient’s circulatory support was gradually weaned off, and he was successfully extubated with a marked improvement in his renal functions. The patient achieved a complete recovery without any long-term sequelae.

**Discussion:**

Cardiogenic shock related to severe rheumatic MV stenosis requires multidisciplinary team management with prompt diagnosis, initiation of the most appropriate mechanical support device (e.g. ECMO or tandem heart), and relief of the MV obstruction. Percutaneous transcatheter mitral commissurotomy can be the preferred option in this setting if the valve is pliable.

**Learning points**

- This case illustrates the importance of rapid diagnosis of valvular heart disease in the setting of cardiogenic shock (CS).
- This case demonstrates the best available choices of mechanical circulatory support devices for CS caused by severe mitral valve stenosis.
- Multidisciplinary team decisions are of extreme paramount to determine the best management pathway of critically ill patients with valvular heart disease.

## Introduction

Cardiogenic shock (CS) is a life-threatening condition where the cardiac output is significantly reduced with haemodynamic instability that compromises the tissue perfusion. Cardiogenic shock carries a high mortality risk of more than 40–50%.^1^ The mainstay of the management is to support the circulation medically and mechanically until the underlying cause has been identified and treated.^2,3^ Although ischaemic heart disease is considered the most common cause of CS,^4^ structural heart disease and valvular dysfunction are considered important causes of CS that necessitate prompt management.^5^ This article highlights a case of severe rheumatic mitral valve (MV) stenosis presenting with tachyarrhythmia and refractory CS successfully supported with extracorporeal membrane oxygenation (ECMO) and treated with percutaneous transvenous mitral commissurotomy (PTMC).

## Summary figure

**Figure:**
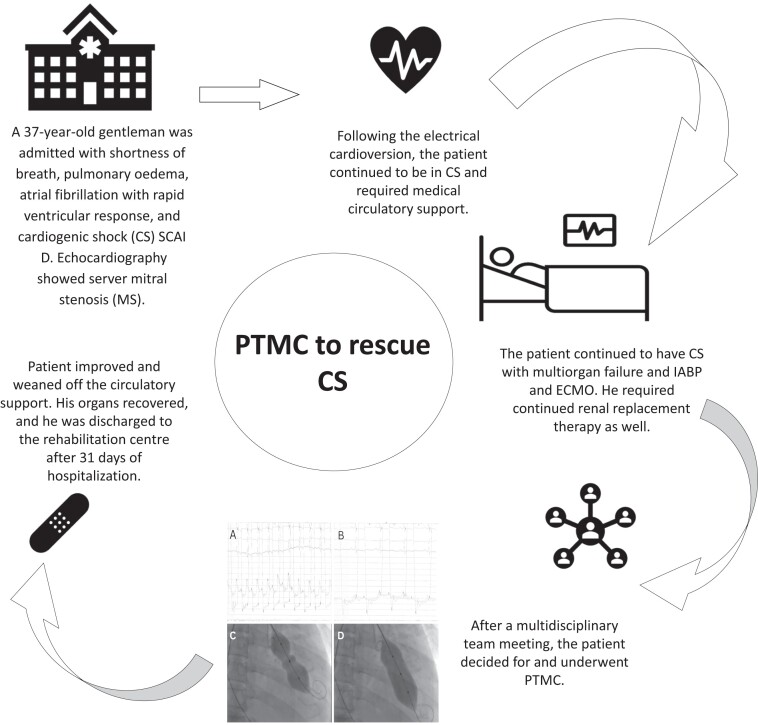


## Case presentation

The emergency medical services brought in a 37-year-old Asian man with an unremarkable past medical history or cardiovascular risk factors because of shortness of breath and palpitations. His symptoms started a few months ago and progressed to the point that he became dyspnoeic at rest. He also had occasional haemoptysis and chest discomfort—no history of chest pain or loss of consciousness.

**Figure 1:**
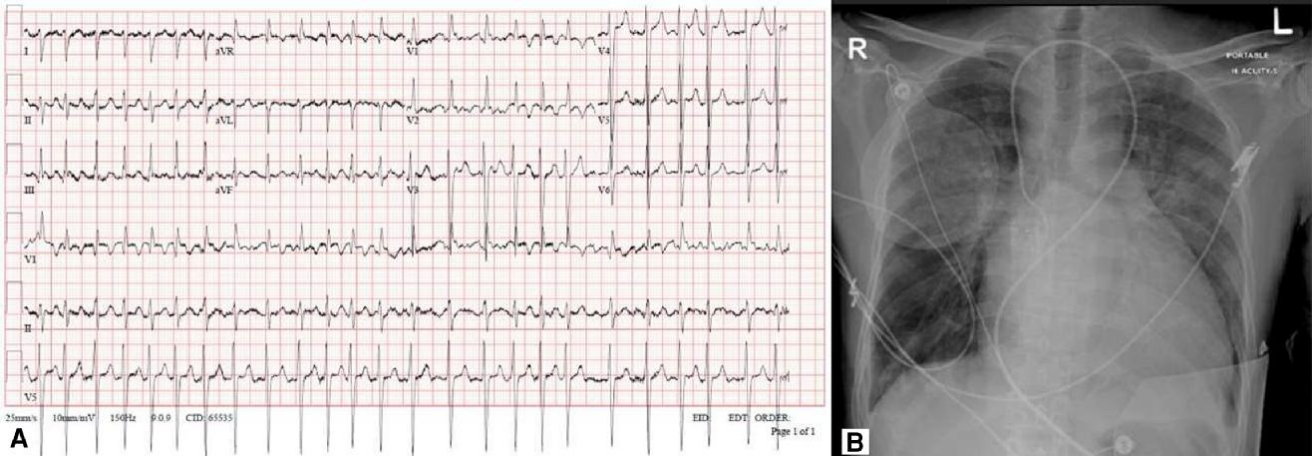
(*A*) Electrocardiogram showing right axis deviation and atrial fibrillation with rapid ventricular response. (*B*) Chest X-ray showing bilateral pulmonary oedema and apparent left atrial enlargement.

On examination, he looked sick, cyanosed, and in respiratory distress. He was afebrile; his respiratory rate was 42 breaths/min, pulse rate was 140 b.p.m. and irregular, blood pressure was 94/77 mmHg, and oxygen saturation was 98% on BiPAP pressure support 6 cm H_2_O, positive end-expiratory pressure 5 cm H_2_O, and 100% FiO_2_. He had diffuse crackles bilaterally, raised jugular venous pressure, and cold extremities with peripheral oedema. His electrocardiogram showed atrial fibrillation (AF) with a rapid ventricular response (*Figure 1A*). Chest X-ray showed cardiomegaly and pulmonary oedema (*Figure 1B*).

As he was in severe respiratory distress, rapid sequence intubation was done with ketamine 100 mg and rocuronium 100 mg. Then, he received 40 mg IV furosemide, 2.5 mcg/kg/min norepinephrine, and 5 mcg/kg/min dopamine for haemodynamic support. He also received two transoesophageal echocardiogram (TEE)-guided direct current shocks of 200 joules without a response and 150 mg amiodarone and 0.5 mg digoxin for the AF, after which sinus rhythm was restored with tachycardia without much improvement in the haemodynamics. The initial laboratory findings showed multiple organ failures with acute kidney injury (AKI), acute liver injury with transaminitis, and a deranged coagulation profile (*Table 1*). He also developed anuric AKI, for which continuous renal replacement therapy (CRRT) was started.

**Table 1:** Initial laboratory results and the end-organ failure

Detail	Value with units			Normal range
WBC	21.9 × 10^3^/uL			10.0–4.0
Hgb	15.2 gm/dL			17.0–13.0
Platelet	269 × 10^3^/uL			400–150
Urea	9.7 mmol/L			7.8–2.5
Creatinine	157 umol/L			106–62
Sodium	126 mmol/L			146–133
Potassium	5.7 mmol/L			5.3–3.5
Bicarbonate	12 mmol/L			29–22
Alk Phos	133 U/L			129–40
ALT	1773 U/L			41–0
AST	3097 U/L			40–0
INR	3.1			1
CRP	130.5 mg/L			0–5
Procalcitonin	4.10 ng/mL			<2
Troponin-T HS	136 ng/L (First set)	300 (Second set)	123 (Third set)	15–3
Pro-BNP	36 659 pg/mL			<300
BG Lac Art-POC	9.90 mmol/L			1.60–0.36
pH	7.11			—
Bicarbonate	12 mmoL/L			22–29
Blood cultures	Negative			—

WBC, white blood cells; Hgb, haemoglobin; Alk Phos, alkaline phosphatase; ALT, alanine transaminase; AST, aspartate aminotransferase; INR, international normalized ratio; CRP, C-reactive protein; HS, highly sensitive; Pro-BNP, pro-B-type natriuretic peptide.

**Figure 2:**
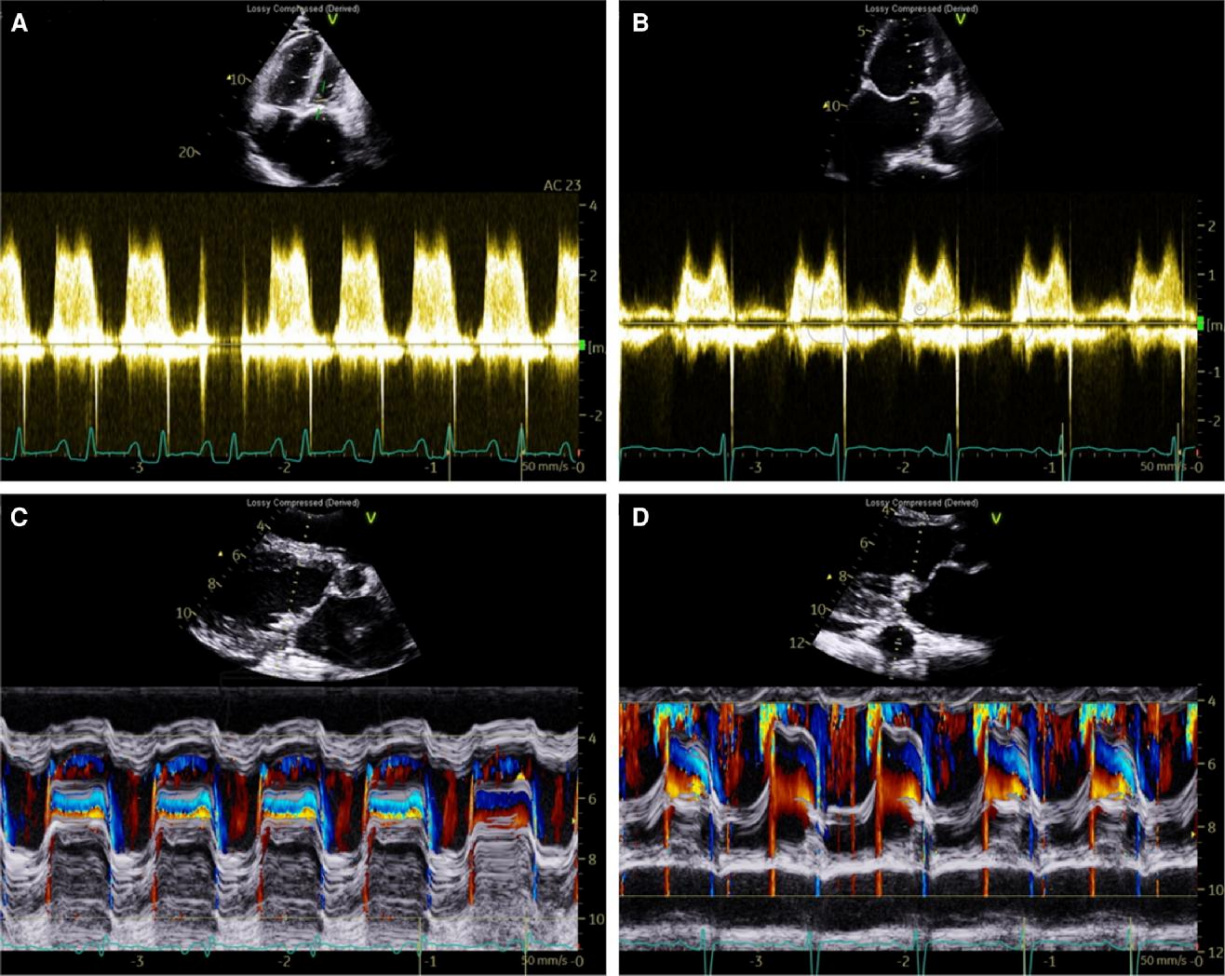
(*A*) Doppler wave transthoracic echocardiogram (TTE) pre-percutaneous transcatheter mitral commissurotomy (PTMC) showing severe mitral valve (MV) stenosis with high mean diastolic gradient and left atrial (LA) dilatation. (*B*) Post-PTMC TTE demonstrating significant improvement in the MV mean diastolic gradient. (*C*) M-mode parasternal long axis (PLAX) pre-PTMC showing severe MV stenosis with restricted movement and opening between the anterior and posterior leaflets and LA dilatation. (*D*) Post-PTMC M-mode PLAX demonstrating significant improvement in the leaflet’s movement and release of the restriction.

Transthoracic echocardiogram (TTE) revealed moderately reduced systolic left ventricular (LV) function, moderate global hypokinesis of LV, an ejection fraction (EF) of 38%, severe rheumatic mitral stenosis (MS) [mitral valve area (MVA) 2D of 0.7 cm^2^, mean diastolic gradient of 17 mmHg, Wilkins score 7], aneurysmal left atrial (LA) dilatation [left atrial end-systolic volume (LAESV): 92 mL/m^2^, left atrial diameter (LAD): 6.5 cm], and moderately reduced right ventricular (RV) function EF 37% (*Figure 2*) (see Supplementary material online, *Videos S1* and *S2*).

The CS persisted and became refractory, Society for Cardiovascular Angiography and Interventions (SCAI) D, he was placed on ECMO and intra-aortic balloon pump (IABP) with prompt improvement in his haemodynamics and a decrease in the requirements of dopamine from 10 to 2 mcg/kg/min and norepinephrine from 3 to 0.1 mcg/kg/min. His Society of Thoracic Surgery (STS) score and EuroScore were 50.1% and 12.1%, respectively, for MV replacement. The invasive haemodynamics showed a pulmonary capillary wedge pressure (PCWP) of 21 mmHg and a mean pulmonary artery pressure of 22 mmHg.

**Figure 3:**
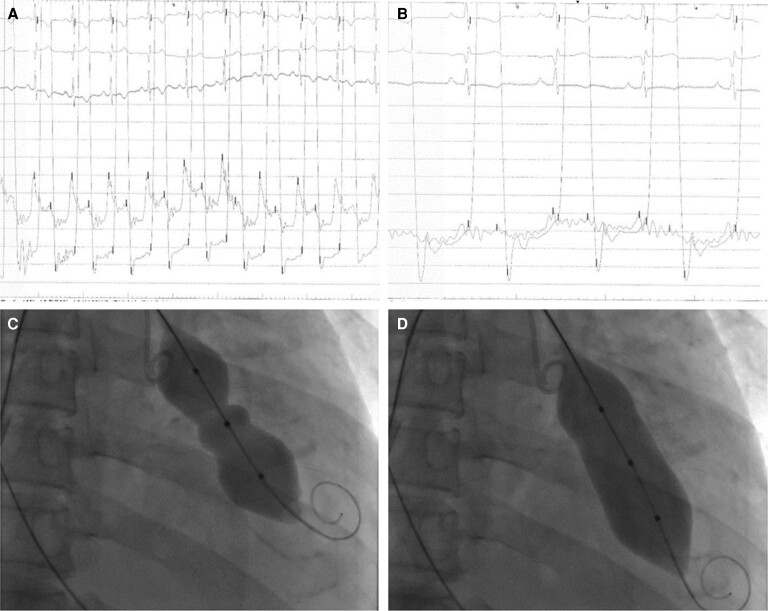
(*A*) Pre-percutaneous transcatheter mitral commissurotomy (PTMC) haemodynamics showing high mean left atrial pressure (LAP) of 17 mmHg with high mean diastolic gradient between the left atrium and left ventricle. (*B*) Post-PTMC haemodynamics showing significant improvement in the mean LAP down to 9 mmHg and almost complete resolution of gradient. (*C*) Still figure showing waist at the balloon during PTMC. (*D*) Still figure showing full inflation and elimination of the stenosis by splitting the mitral valve commissural fusion.

On Day 5, the patient underwent PTMC after the multidisciplinary team discussion determined that he had extreme surgical risk and his valve was pliable for percutaneous intervention. The procedure was performed with transoesophageal guidance. Due to the shortage of dedicated PTMC balloons, the procedure was performed with a 26 mm × 40 mm Numed balloon over Safari wire after obtaining transseptal access using a steerable guide sheath. After one balloon inflation, his mean LA pressure (LAP) and mean diastolic MV improved from 17 to 9 mmHg and from 9 to 2 mmHg, respectively (*Figure 3*), without significant mitral regurgitation (MR). A follow-up TTE on the same day demonstrated MVA 2D improvement to 1.9 cm^2^ and tricuspid annular plane systolic excursion (TAPSE) improvement to 1.2 cm, indicating residual mild RV function impairment and LV EF improvement to 48%. His haemodynamics improved afterward, and he was weaned off ECMO, decannulated on Day 6, and extubated on Day 14. His CRRT was switched to intermittent haemodialysis, and eventually, his renal function fully recovered on Day 23, and he did not require haemodialysis or diuresis afterward. He was then transferred to the rehabilitation centre for physical therapy as he developed critical care myopathy during his hospital stay. After 2 months of his stay in the rehabilitation centre, the patient was discharged home with normal cognitive function and independent mobility (ambulation and stair climbing). He was discharged on bisoprolol (dose) for rate control, warfarin for valvular AF to target international normalized ratio (INR) level of 2–3, and amlodipine (dose) for hypertension and planned to further optimize his heart failure medications during his follow-up clinical visits. The patient will be followed up in the structural cardiology and heart failure clinics with repeated transthoracic echocardiography and further workup for a reduced EF if it persisted.

## Discussion

Cardiogenic shock is a cardiac emergency that requires immediate management through circulatory support and immediately identifying and correcting the major cause. Structural heart disease and valvopathies are not uncommon causes of CS. Although aortic stenosis is the most common valvopathy, followed by mitral and aortic regurgitation and MS, any form of severe valvular lesions might induce a CS.^6^ Valvular lesions tend to have a chronic clinical presentation; however, they might be manifested critically if pre-existing LV dysfunction and coronary artery disease are present or, as in this case, the development of superimposing cardiac arrhythmia leading to haemodynamic instability.^7,8^ Nevertheless, valvular lesions should be managed once they are the CS culprit.

The typical management of CS caused by valvopathies requires inotropic and vasopressors and, in advanced stages, adjunctive mechanical circulatory support as a bridge until further intervention is sought. As there are different types of mechanical circulatory supporting devices, the selection of an appropriate device hinges upon various factors, including the availability of options and the expertise of the clinical team.^9^ To facilitate this decision-making process, Villablanca et al.^10^ have proposed a pragmatic algorithm and ischaemic flow chart that consider both the haemodynamic impact of the devices and the underlying valvular pathologies, thereby enabling tailored device selection for each specific valvular lesion. The general principles stated by the European Acute Cardiovascular Care Association are to minimize the use of catecholamines and inotropes, to not use aortic pump routinely, and to consider the RV support when there is RV failure.^11^ In severe MS, the preferred mechanical circulatory support (MCS) is veno-arterial (VA) ECMO or LA–VA ECMO.^10^ This would be a bridgeport for the definitive management, which is a valvular repair, and as the patients are critically ill, minimally invasive procedures are considered first. In severe MS, PTMC has been reported to alleviate patients with severe pulmonary oedema, intractable heart failure, and CS. It is regarded as a feasible and lifesaving procedure.^12,13^

In this case, ECMO was chosen to provide both respiratory and circulatory support. Intra-aortic balloon pump (IABP) was added to help reduce the afterload and reduce the risk of LV overdistention. Alternatively, LA–VA ECMO or tandem heart with external oxygenation membrane could have been considered in this case. Meanwhile, definitive management was sought in a multidisciplinary team fashion involving cardiac surgeons, cardiac anaesthesia, and interventional cardiologists. As the patient was fragile with unstable haemodynamics and the valve was pliable for percutaneous intervention, catheter-based management was determined for him with excellent outcomes.

## Conclusion

Cardiogenic shock related to severe rheumatic MV stenosis requires multidisciplinary team management with prompt diagnosis, initiation of the most appropriate mechanical support device (e.g. ECMO or tandem heart), and relief of the MV obstruction. Percutaneous transcatheter mitral commissurotomy can be the preferred option in this setting if the valve is pliable.

## Lead author biography


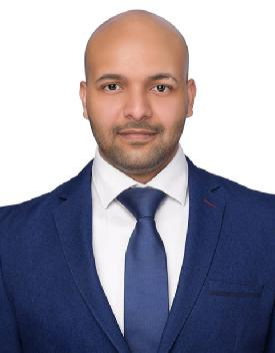
As a future cardiologist, Dr Ashraf Ahmed has a burning desire to learn as much as possible about the field of cardiology through clinical research. He hopes to do this through connecting with like-minded professionals and mentors. Dr Ahmed is a POCUS coach at Hamad Medical Corporation, Affiliated with Weil Cornell Medicine in Qatar. He also achieved the Paul Dudley White International Award at the AHA Conference 2021. In 2022, Harvard T.H. Chan School of Public Health recognized him as an outstanding Teaching Assistant-I for the Principle and Practice of Clinical Research Course.

## Supplementary material

Supplementary material is available at *European Heart Journal – Case Reports* online.

## Supplementary Material

ytad553_Supplementary_Data

## Data Availability

All the data supporting this article and related to this case report are available with the authors.
